# Supplementary material for: Adenosine Methylation Level of miR-125a-5p Promotes Anti-PD-1 Therapy Escape through the Regulation of IGSF11/VSIG3 Expression
Source: Cancers (Basel). 2023 Jun 14;15(12):3188. doi: 10.3390/cancers15123188 (PMC10296663; doi:10.3390/cancers15123188)
Supplement: Supplementary file 1 [file cancers-15-03188-s001.zip › cancers-2355547-supplementary.pdf]

A

	1	2	3	4	5	6	7	8	9	10	11	12	13	14	15	16	17	18	19	20	21	22	23	24
**miR-125a-5p**	u	c	c	c	u	g	a	g	a	c	c	c	u	u	u	a	a	c	c	u	g	u	g	a
							D	R	A	C	H				D	R	A	C	H					
**miR-181c-5p**	a	a	c	a	u	u	c	a	a	c	c	u	g	u	c	g	g	u	g	a	g	u		
															c	g								

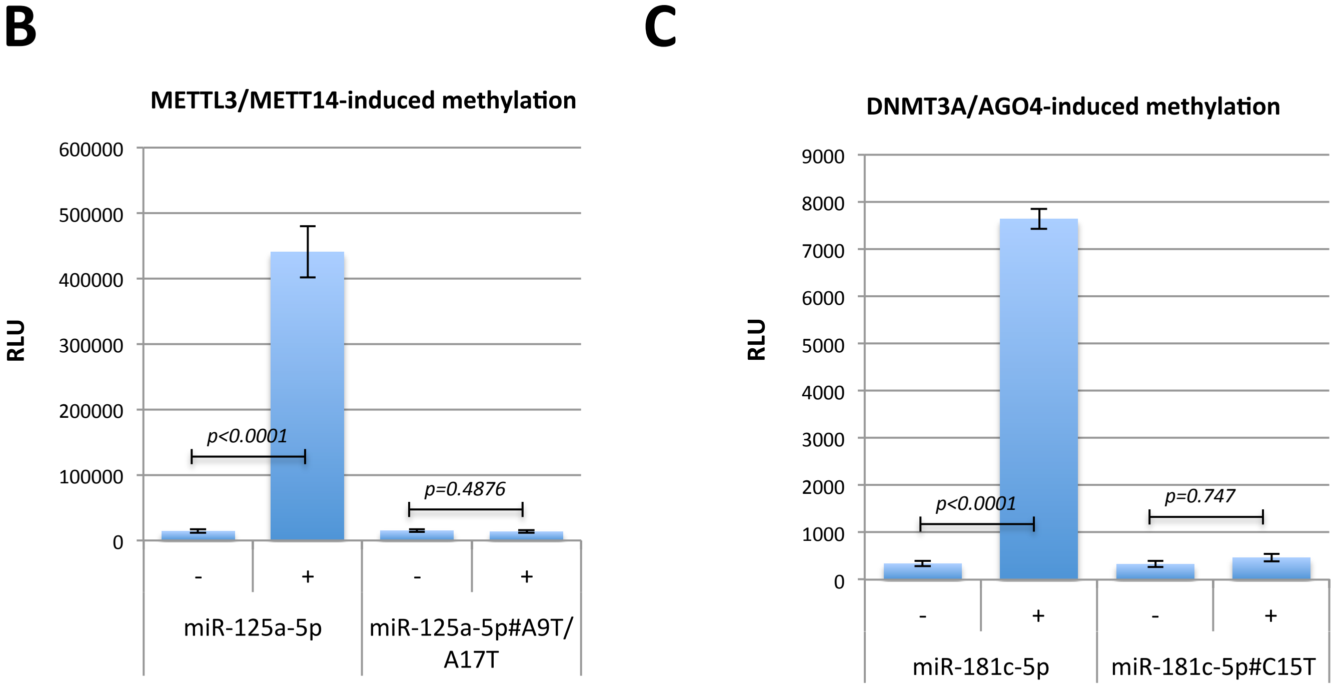

Supplementary Figure S1.

Identification of methylated nucleotides in miRNAs.

A. position of nucleotides identified as methylatable adenosine and cytosine in miR-125a-5p and miR-181c-3p.

B and C. This experiment was performed using the MTase-Glo Methyltransferase Assay Kit (Promega). ThemiRNA (2 µM) was incubated with i) 0.2 µM of DNMT3A/AGO4 complex (Active Motif, France) in a reaction buffer containing 20 mM Tris–HCl (pH 8.0), 50 mM NaCl, 1 mM EDTA, 1 mM DTT, 5% glycerol, 0.1 mg/ml bovine serum albumin (BSA) and 10 µM SAM at 37°C for 1 h; or ii) 0.5 µM of METTL3 / METTL14 complex (Active motif, France) in a reaction buffer containing 50 mM Tris-HCl pH 8.6,0.02% Triton X-100, 2 mM MgCl2, 1 mM TCEP and 10 µM SAM at 21-23° for 1h. The reaction was stopped by addition of 1 µl 0.5% trifluoroacetic acid (TFA). After 5 min of incubation with TFA, 5×MTase-Glo Reagent was added, mixed well and incubated at room temperature for 30 min. Finally, MTase-Glo Detection was added and luminescence were read after another 30 min incubation.

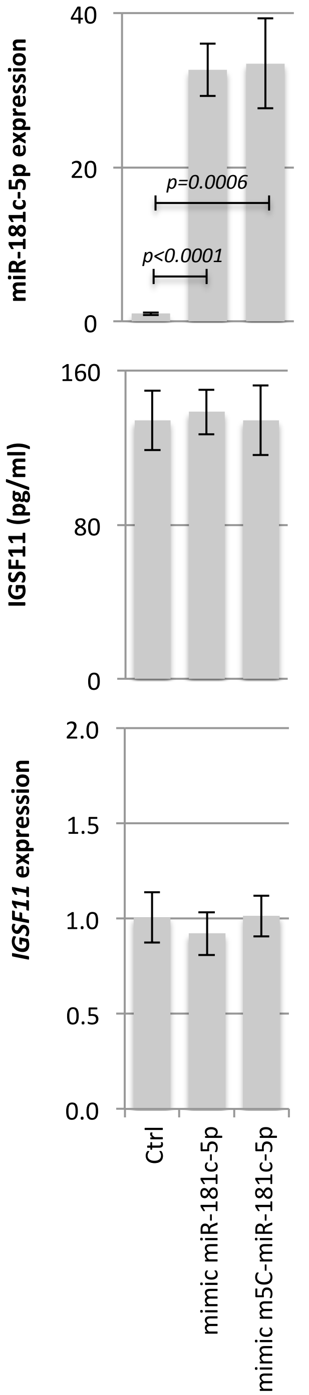

Supplementary Figure S2

Impact of the mimic-miR-181c-5p and mimic-m6a-miR-181c-5p on the expression of ISGF11. Briefly, 6×10^5 cells were seeded in each well of 6-well plates. Transfection was performed using HiPerFect Transfection Reagents (Qiagen#301705, France) and 10 ng miRNA. For miR control, transfection control (HiPerfect Transfection Reagent only) and an oligo (miScript Inhibitor Negative Control; Qiagen, France) had been used. 48h after the transfection, cells are recovered to perform miRNA and protein extractions as described in this manuscript. Expression of miRNA-181c-5p was performed by RT-qPCR and IGSF11 by ELISA, as described in this manuscript. Mimic m5C-miR-181c-5p was methylated at cytosine in position 15.

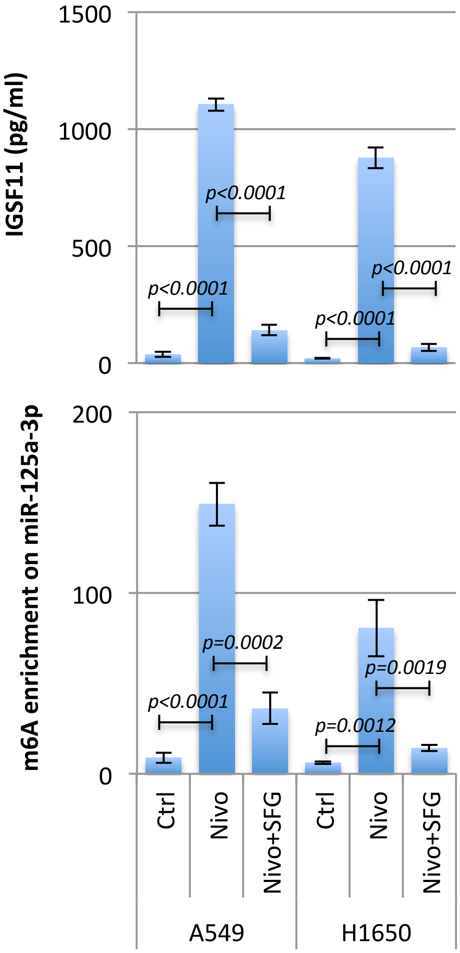

Supplementary Figure S3

Impact of Nivolumab on two other lung cancer cell lines: A549 and H1650.

The Nivolumab impact on the adenosine methylation level and the IGSF11 expression level was also investigated in two other lung cancer cell lines: A549 and H1650.

RIP and ELISA indicated that the Nivolumab increased the adenosine methylation level of miR-125a-5p and the ISGF11 expression level (similarly to the observation made in H1975 cells).

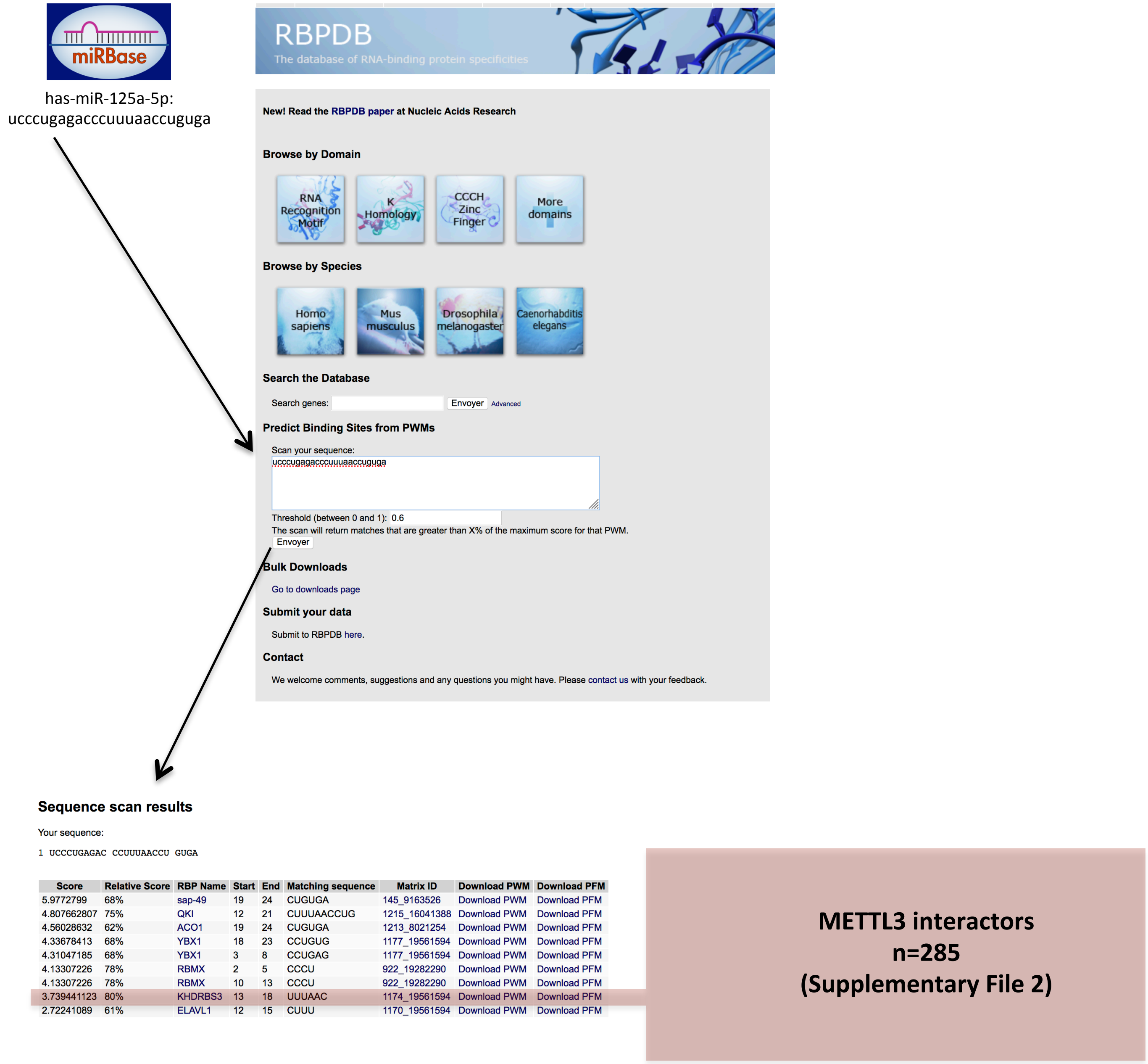

Score	Relative Score	RBP Name	Start	End	Matching sequence	Matrix ID	Download PWM	Download PFM
5.9772799	68%	sap-49	19	24	CUGUGA	145_9163526	Download PWM	Download PFM
4.807662807	75%	QKI	12	21	CUUUAACCUG	1215_16041388	Download PWM	Download PFM
4.56028632	62%	ACO1	19	24	CUGUGA	1213_8021254	Download PWM	Download PFM
4.33678413	68%	YBX1	18	23	CCUGUG	1177_19561594	Download PWM	Download PFM
4.31047185	68%	YBX1	3	8	CCUGAG	1177_19561594	Download PWM	Download PFM
4.13307226	78%	RBMX	2	5	CCCU	922_19282290	Download PWM	Download PFM
4.13307226	78%	RBMX	10	13	CCCU	922_19282290	Download PWM	Download PFM
3.739441123	80%	KHDRBS3	13	18	UUUAAC	1174_19561594	Download PWM	Download PFM
2.72241089	61%	ELAVL1	12	15	CUUU	1170_19561594	Download PWM	Download PFM

Supplementary Figure S4

Schematic representation of the *in silico* analysis performed with the RBPDB site to identify KHDRBS3 as binding to miR-125a-5p.

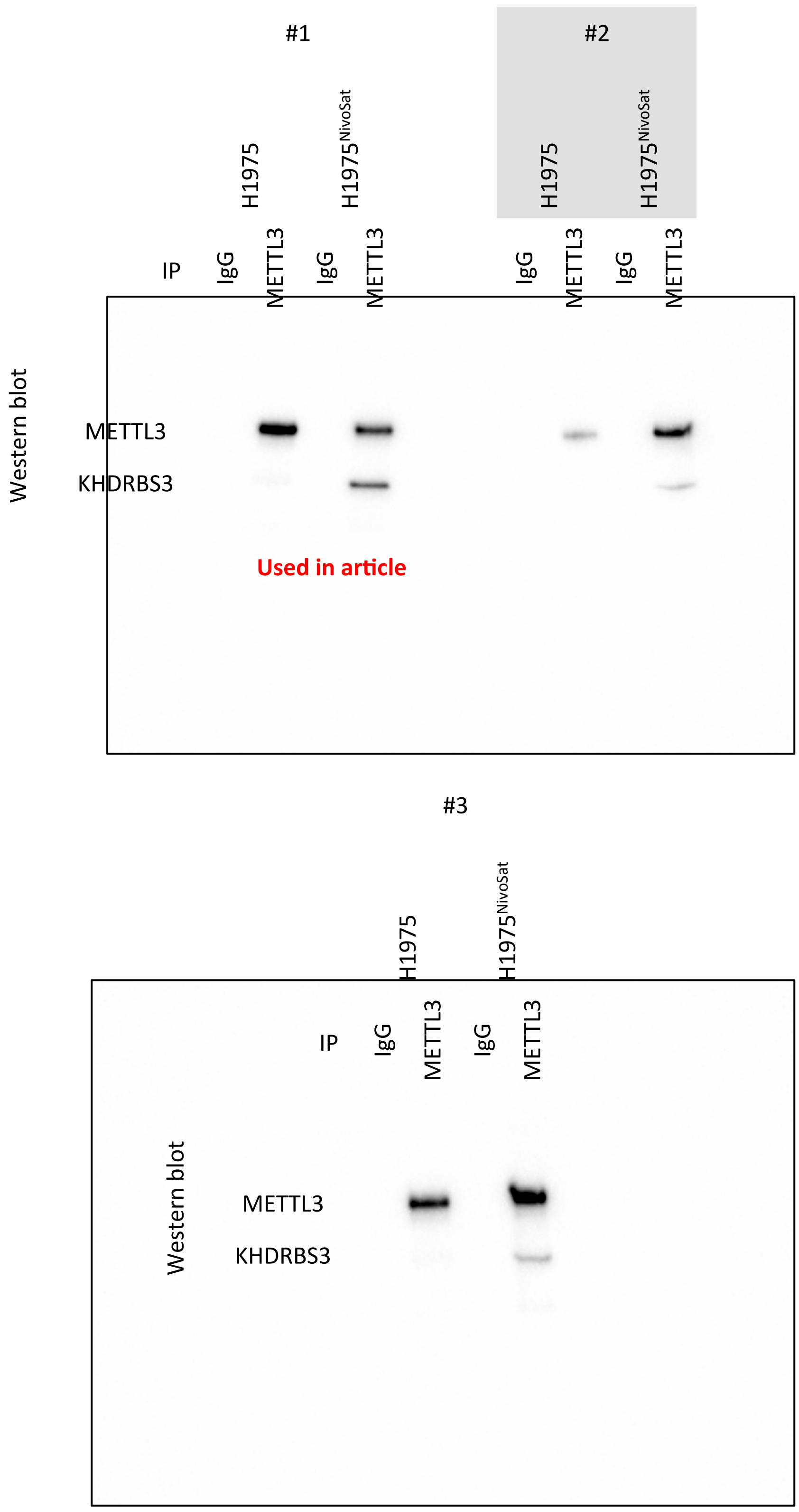

Supplementary Figure S5.

Illustration of the three independent co-immunoprecipitations performed.

The images shown in this figure are from western blot membrane revelations made with the ChemiDoc (Bio-Rad) in "autoexposure" mode and the use of the ECL-Clarity kit (Bio-Rad).

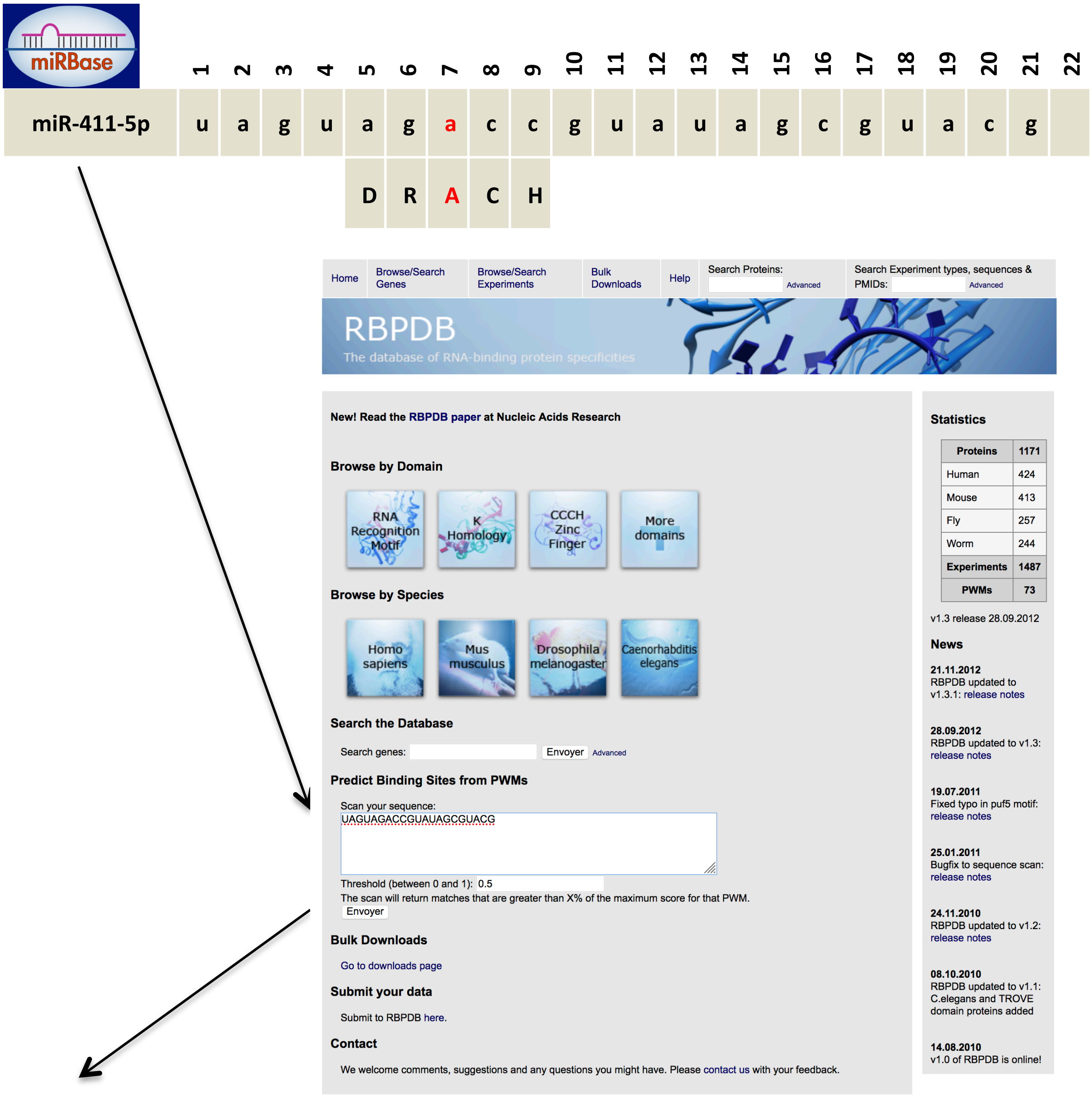

Sequence scan results

Your sequence:

```
1 UAGUAGACCG UAUAGCGUAC G
```

Score	Relative Score	RBP Name	Start	End	Matching sequence	Matrix ID	Download PWM	Download PFM
5.36340303	86%	YTHDC1	15	20	GCGUAC	969_20167602	Download PWM	Download PFM
2.57730567	53%	RBM4	14	17	AGCG	1172_19561594	Download PWM	Download PFM

Supplementary Figure S6

Schematic representation of the *in silico* analysis performed with the RBPDB site to identify KHDRBS3 as binding to miR-125a-5p.

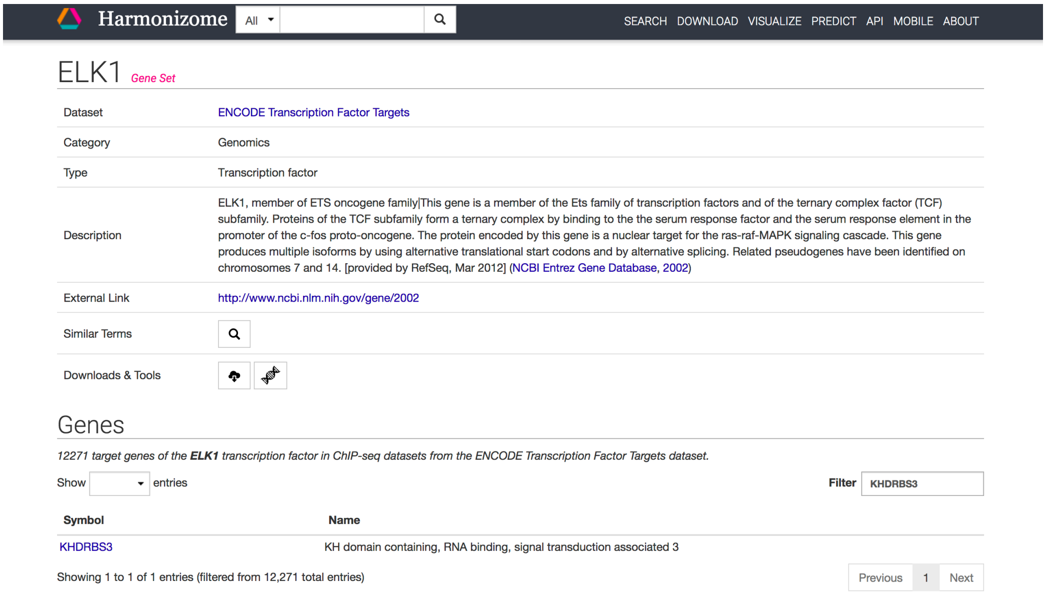

Supplementary Figure S7

Screenshot of the use of the Harmonizome site.

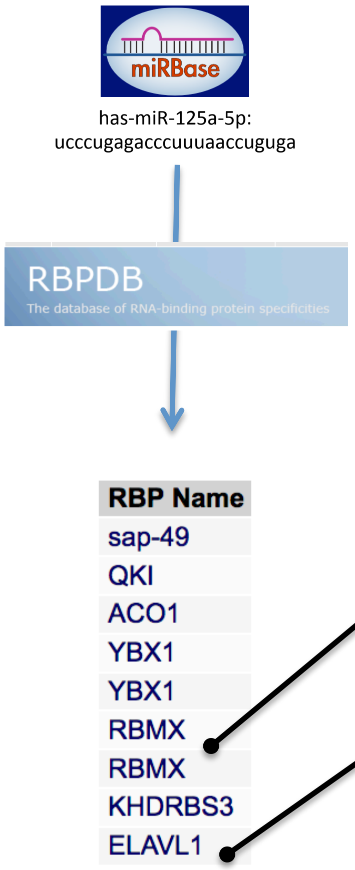

List of m6A-binding proteins

Name	References
YTHDF1	Zhao, Y., Shi, Y., Shen, H. *et al.* m6A-binding proteins: the emerging crucial performers in epigenetics. *J Hematol Oncol* **13**, 35 (2020). https://doi.org/10.1186/s13045-020-00872-8
YTHDF2	
YTHDF3	
YTHDC1	
YTHDC2	
HNRNPA2B1	
HNRNPC	
**HNRNPG (RBMX)**	
IGF2BP1	
IGF2BP2	
IGF2BP3	
**HuR (ELAVL1)**	
SND1	
RBM45	Choi et la. Cell Rep. 2022. PMID: 36044854
YTHDF1	Zhang et Hamada Front. Genet. 2021
YTHDF2	
YTHDF3	
RBM15	
RBM15B	
YTHDC1	
EIF3D	
NOP58	
HNRNPH1	
NUDT21	
FMR1	
DDX3X	
EIF3A	
CPSF6	
CPSF7	
RBM3	
CREBBP	
SRSF2	
SRSF1	
CPSF6	
CIRBP	

Supplementary Figure S8.

Schematic representation of the *in silico* analysis performed to identify the "m6A-binding proteins" having the ability to bind miR-125a-5p according to the use of the RBPDB website.

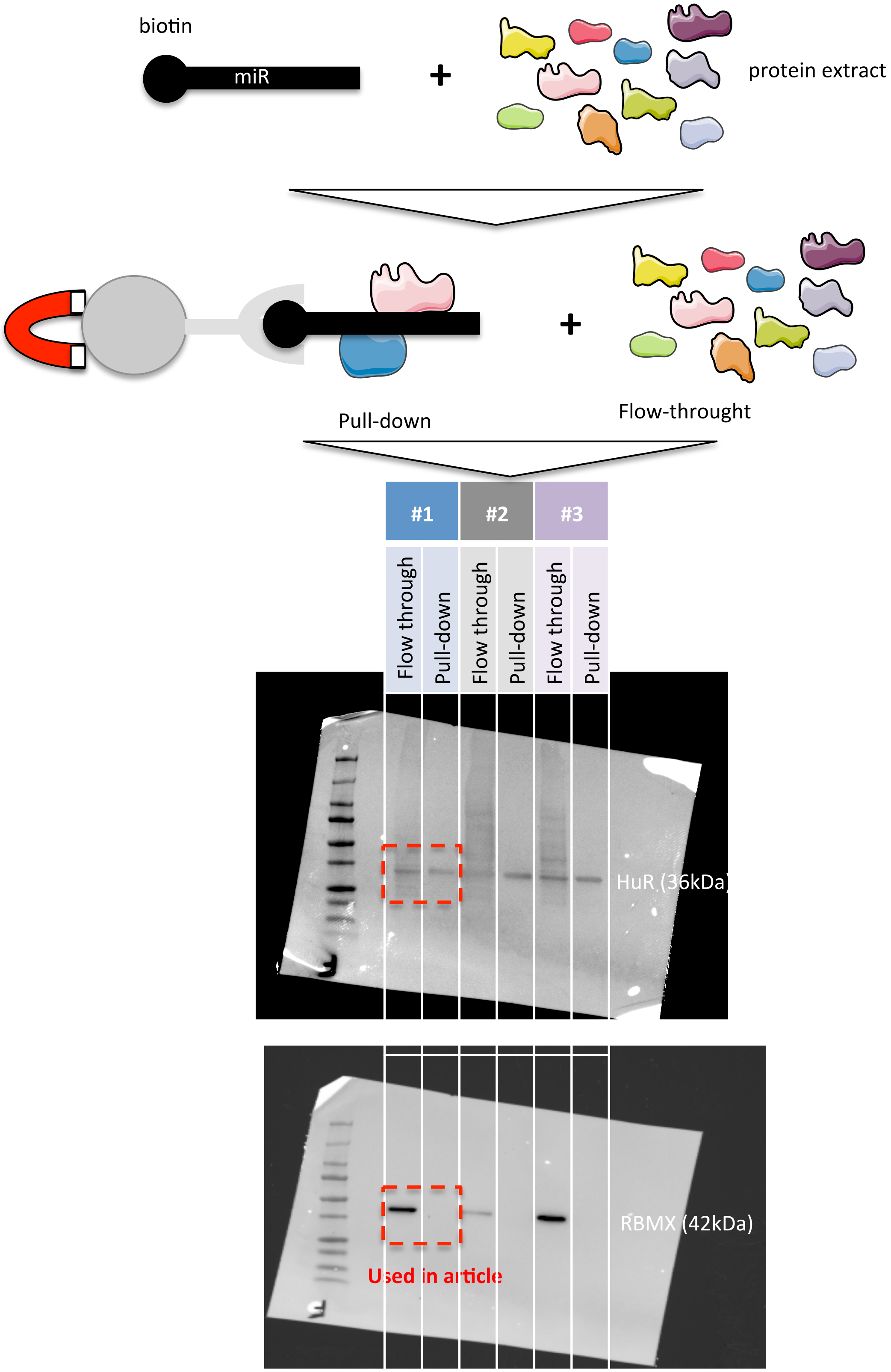

Supplementary Figure S9.

Illustration of the miRNA pull-down assay.

The images shown in this figure are from western blot membrane revelations made with the ChemiDoc (Bio-Rad) in "autoexposure" mode and the use of the ECL-Clarity kit (Bio-Rad). #1, #2 and #3 represente the triplicate of experiments.

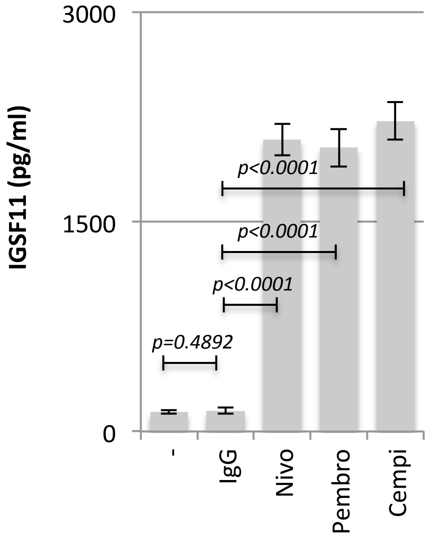

Supplementary Figure S10

Impact of Pembrolizumab and Cemiplimab on the IGSF11 expression.

The impact of Pembrolizumab (Pembro, 0.1 µg/ml) and Cemiplimab (Cemip, 0.1 µg/ml) on IGSF11 expression level was compared to the one of Nivolumab (Nivo) according with the experimental design shown in Figure 1B. IgG antibody (0.1 mg/ml) was used as control. Analysis of IGSF11 expression was performed using ELISA (#LS-F65302, LSBio, US).

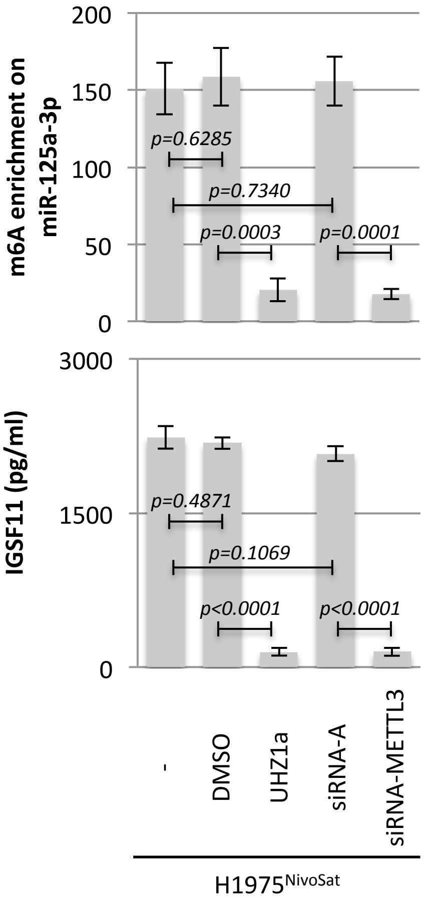

Supplementary Figure S11

Impact of chemical and biological inhibition of METTL3 on the adenosine methylation level of miR-125a-3p and ISGF11 expression. H1975 cells were treated with Nivolumab as described in Figure 1B. Then, UHZ1a (30mM, 48h, #HY-134673A, MedChemExpress, Europe) and siRNA-METTL3 (50pmol, #sc-92172, Santa Cruz, France) were used to chemically and biologically inhibit METTL3 in these cells. DMSO and siRNA-A (#sc94636, Santa Cruz, France) were used as control. HIPerfect Transfection Reagent kit (#301705, Qiagen, France) were used to perform siRNA transfection according with the manufacturer's instructions. m6A-RIP and ELISA were then performed as already described in this manuscript.
